# The prognostic value of positron emission tomography/magnetic resonance imaging in predicting survival in patients with adenocarcinoma of the esophagogastric junction

**DOI:** 10.1007/s12149-025-02058-z

**Published:** 2025-05-17

**Authors:** Gustav Holm Schæbel, Helle Hjorth Johannesen, Johan Löfgren, Henrik Gutte, Lene Bæksgaard, Michael Patrick Achiam, Mohamed Belmouhand

**Affiliations:** 1 Department of Surgery and Transplantation, Rigshospitalet, University of Copenhagen, Blegdamsvej 9, 2100 Copenhagen, Denmark; 2 Department of Clinical Physiology, Nuclear Medicine and PET, Rigshospitalet, University of Copenhagen, Blegdamsvej 9, 2100 Copenhagen, Denmark; 3 Department of Oncology, Rigshospitalet, University of Copenhagen, Blegdamsvej 9, 2100 Copenhagen, Denmark; 4 Department of Ophthalmology, Rigshospitalet, University of Copenhagen, Valdemar Hansens Vej 1, 23, 2600 Glostrup, Denmark

**Keywords:** Positron-emission tomography, Magnetic resonance imaging, Multimodal imaging, Esophageal neoplasms, Response evaluation criteria in solid tumors, Survival

## Abstract

**Introduction:**

In recent years, the utility of positron emission tomography/magnetic resonance imaging (PET/MRI) has become increasingly significant in diagnostic settings. This study provides a five-year follow-up on a previous pilot study that demonstrated the feasibility of PET/MRI in predicting the resectability of adenocarcinoma of the esophagogastric junction (AEG). We aimed to evaluate whether this imaging modality could further serve as a prognostic tool for survival in AEG patients.

**Methods:**

A total of 22 patients were included in the initial pilot study, with 17 of them undergoing surgery. All patients underwent three series of neo-adjuvant chemotherapy (NT). This follow-up study retrospectively analyzed the correlation between the apparent diffusion coefficient (ADC) and standard uptake value (SUV) measurements of the primary tumor from the original study with overall survival and recurrence. ADC and SUV values were measured prior to initiation of NT, and again 17–21 days into the first cycle of NT-administration, and the differences between the scans were calculated as ∆SUV_max_, ∆ADC_b0_, and ∆ADC_b50_. Early treatment response was assessed using the Response Evaluation Criteria In Solid Tumors (RECIST). Binary logistic regression was employed to evaluate the predictive values of ADC and SUV parameters, and receiver operating characteristic (ROC) curves were generated to determine sensitivity, specificity, and area under the curve (AUC).

**Results:**

As of January 7, 2022, 8 of the 22 patients were still alive. The AUC was calculated to assess the association of imaging parameters with long-term survival: ∆SUV_max_: AUC = 0.74, sensitivity, 87.5%, specificity 62.5% (*p* = 0.037). ∆ADC_b0_: AUC = 0.62, sensitivity 85.7%, specificity 57.1% (*p* = 0.400). ∆ADC_b50_: AUC = 0.78, sensitivity 78.6%, specificity 85.7% (*p* = 0.011). Combining all three parameters yielded an AUC of 0.81, with a sensitivity of 78.6% and a specificity of 85.7% (*p* = 0.002). The results for individual measurements were: SUV_max_(pre-NT): AUC = 0.56, sensitivity 78.6%, specificity 50% (*p* = 0.646). SUV_max_(post-NT): AUC = 0.81, sensitivity 85.7%, specificity 87.5% (*p* = 0.002). ADC_b0_(pre-NT): AUC = 0.55, sensitivity 71.4%, specificity 62.5% (*p* = 0.682). ADC_b0_(post-NT): AUC = 0.63, sensitivity 78.6%, specificity 57.1% (*p* = 0.339). ADC_b50_(pre-NT): AUC = 0.51, sensitivity 85.7%, specificity 37.5% (*p* = 0.952). ADC_b50_(post-NT): AUC = 0.63, sensitivity 42.9%, specificity 100% (*p* = 0.279). No significant correlation was found between RECIST group and survival status (*p* = 0.15).

**Conclusion:**

Our results indicate that PET/MRI is feasible for predicting long-term survival in AEG patients. The highest AUCs were achieved when combining SUV and ADC parameters, and when using post-NT SUV_max_ alone.

## Introduction

The prognosis for patients diagnosed with adenocarcinoma of the esophagogastric junction (AEG) is dismal, and up towards two thirds cannot be offered curative treatment—and even those undergoing curative treatment face high recurrence rates and poor overall survival [1]. Currently, no reliable screening methods for AEG exist, and the disease typically presents symptomatically at an advanced stage [2].

In Denmark, the standard treatment for patients with resectable AEG usually involves eight weeks of neoadjuvant chemotherapy (NT), followed by surgical resection of the cancer and an additional eight weeks of adjuvant chemotherapy [1]. Data from our institute indicates a five-year recurrence rate of 35% among patients who underwent curative surgery [3], with some international rates reaching as high as 60% [4, 5]. The median time to recurrence was 17 months [3].

Identifying patients who will not benefit from surgical cancer removal remains a significant challenge. Positron emission tomography/computed tomography (PET/CT) is currently the preferred tool for assessment and staging worldwide [6]. In recent years, the combined PET/MRI modality has gained diagnostic value in various medical fields, showing particular promise in oncology [7, 8]. For esophageal cancer, PET/MRI has demonstrated comparable results to PET/CT in TNM staging and potentially higher sensitivity for detecting metastases [9, 10]. However, its prognostic value for AEG remains unexplored.

Our department previously published a study indicating that PET/MRI could predict resectability in patients with AEG [11]. This five-year follow-up study aims to investigate whether PET/MRI can be used to identify long-term survivors (> 5 years) based on preoperative scans.

## Methods

### Study design

This is a retrospective cohort study investigating the prognostic value of simultaneous PET/MRI, correlating the difference in (NT)-response among responders and non-responders to survival, investigating the difference in survival time, and assessing the predictive performance of the modality’s imaging biomarkers using receiver operating characteristics (ROC) curves. The original study protocol can be found on clinicaltrials.gov, ID NCT02433301. This study is reported according to the Standards for Reporting Diagnostic accuracy studies (STARD) 2015 guideline [12].

### Patients

Patients with biopsy-proven AEG were included at Rigshospitalet, Denmark, from April 2015 to March 2016. Only patients above 18 years of age diagnosed with untreated, biopsy-proven resectable adenocarcinoma of the esophagogastric junction (Siewert types I/II), who were suitable for neoadjuvant therapy and had no contraindications for PET/MRI or history of diabetes, were deemed eligible. Resectability was assessed at a multidisciplinary team conference based on initial examinations (endoscopy with biopsy, diagnostic laparoscopy, and PET/CT). The study was approved by the local regional ethics committee (ID: H-1-2014-076), and all patients gave their oral and written informed consent prior to enrolment. A baseline scan was made prior to NT treatment, and an evaluation scan was performed before initiating the second cycle of NT, 17–21 days after the first cycle. The predefined inclusion criteria, NT protocol, 18 F-FDG PET protocol, and MRI protocol are all described in detail in the pilot study [11]. All patients received an NT regimen of epirubicin, cisplatin, and infused fluorouracil as described in the MAGIC trial [13].

In this study, all patients had their medical history reviewed, focusing on survival status until January 7, 2022. At this point, at least five years had passed since the date of the baseline scan for all patients.

### ^18^F-FDG PET/MR imaging

All scans were performed using a hybrid PET/MRI scanner (Biograph mMr, Siemens AG, Erlangen, Germany).

For the PET examination, patients were given an 18-F FDG injection (4 Mbq/kg) after at least 6 h of fasting. Prior to injection, blood glucose levels were measured to ensure normal values. An hour after injection, a six-minute static emission scan was acquired from mid-thoracic to upper gastrointestinal level. Using 3D OP-OSEM, images were reconstructed with four iterations and 21 subsets on a 344 × 344 matrix. A Gaussian post-filter of 4 mm was applied. Standard uptake values (SUV) were normalized for injected FDG dose and the patient’s body weight. A nuclear medicine consultant evaluated and analyzed PET scans using SyngoVia software (Siemens Medical Solutions). The maximum SUV value (SUV_max_) within the tumor region was measured on the baseline scan, and the same region was measured on the evaluation scan. To make sure the maximum SUV value in the tumor region was indeed within the tumor region, the MRI acquired simultaneously was reviewed in the process. The difference between the baseline and evaluation scan was calculated using percentages (∆SUV_max_ (%)) by dividing the difference between baseline SUV_max_ and evaluation SUV_max_ with baseline SUV_max_.

For the MRI scans, 20 mg of hyoscinbutylbromid was administered as an intramuscular injection five minutes prior to each examination to minimize the risk of peristaltic artifacts. The imaging protocol consisted of T2-weighted Half Fourier Acquisition Single Shot Turbo Spin Echo (HASTE) sequences in sagittal, transversal, and coronal orientation, serving as additional scout imaging. For tumor characterization, transversal-oriented HASTE and two echo planar Diffusion-Weighted Imaging (DWI) with *b*-values of 0, 50, 400, and 800 s/mm^2^ were performed. DWI was triggered by respiration using an MR navigator on the diaphragm, whereas T2-weighted imaging was scanned in breath-hold. A radiology consultant evaluated and analyzed the MR images using Mirada XD viewer software, version 1-2-0-59 (Mirada Medical, Oxford, UK). Regions of interest (ROI) were drawn freehand on three consecutive slices outlining the tumor and using high B-values (primarily b400). If impractical to technically achieve three slices of sufficient quality, only one or two consecutive slices were used. Two apparent diffusion coefficient (ADC) maps were calculated, the first using b0, b400, and b800 s/mm^2^ (ADC0) and the second using b50, b400, and b800 s/mm^2^ (ADC50). In both cases, the mean ADC values in the ROI were used. This was done to allow for comparison with measurements otherwise found in the literature. As with the SUV from the PET scans, the percentage differences between baseline and the evaluation scans were calculated (∆ADC_b0_ (%) and ∆ADC_b50_ (%)). Early response to treatment was evaluated using the Response Evaluation Criteria In Solid Tumors (RECIST) guideline version 1.1 [14].

### Statistics

IBM SPSS statistics version 25 was used for statistical analyses. Non-parametric statistics were applied throughout. Five-year survival was calculated and plotted using the Kaplan–Meier method. ADC and SUV parameters were combined using binary logistic regression to generate predicted probability values, which were then used to construct receiver operating characteristics (ROC) curves and evaluate combined prognostic performance. An optimal cutoff value was chosen (closest to the (0,1)-point) with the corresponding sensitivity, specificity, and area under the curve (AUC). The association with survival across RECIST categories was calculated with a Fisher’s exact test. Median values were calculated for pre- and post-NT SUV_max_, ADC_b0_, and ADC_b50_, and the differences between the baseline scan and the evaluation scan. These values and differences were compared between survivors and non-survivors using the Mann–Whitney *U* test. A two-sided *p*-value below 0.05 was considered statistically significant. Patients who were found unresectable after NT were also included in the analyses.

## Results

A total of 22 patients were enrolled in the study (Table 1). As of January 7 th, 2022, eight patients were still alive, and 14 had died. The overall median survival time for all participants was 25 months. Among the 14 deceased, five had not undergone surgery due to disease progression during NT. No patients were lost to follow-up. The cause of death was cancer-related in all but two cases, where it was unspecified in the available records. None of the survivors experienced AEG recurrence during the follow-up period. The median age of the survivors was 68 years, compared with 62 years for the deceased at the time of the baseline scan, though this difference was not statistically significant (*p* = 0.616).

**Table 1:** Demographic data

	Survivors *N* = 8	Non-survivors *N* = 14	*p* value
Gender			0.262
Male	8	12	
Female	0	2	
Age in years, median (IQR^a^)	68 (58–69)	62 (58–70)	0.616
BMI, median (IQR)	24.50 (23.68–27.38)	24.50 (21.05–28.20)	0.868
Smoking^b^			0.430
Smoker	4	10	
Non-smoker	4	4	
Alcohol consumption^c^			0.402
< 10 units/week	6	8	
> 10 units/week	2	6	
ASA score^d^			0.202
1	3	1	
2	4	11	
3	1	2	
cT-stage			0.309
1	1	0	
2	3	2	
3	3	10	
4	1	2	
cN-stage			0.353
0	6	5	
1	1	6	
2	0	1	
3	1	2	
ypT-stage			0.004*
0	4	0	
1	2	0	
2	1	0	
3	1	9	
Not assessed^e^	0	5	
ypN-stage			0.052
0	7	2	
1	1	3	
2	0	2	
3	0	2	
Not assessed^e^	0	5	

^a^IQR: interquartile range

^b^Smoker defined as active/history of use of tobacco corresponding to > 1 pack-year (≥ 20 cigarettes/day for 365 consecutive days)

^c^According to national health recommendations, below/above

10 units per week for both men and women

^d^American Society of Anesthesiologists score

^e^5 patients had progression of disease and did not undergo surgery, why pT- and pN-stage was not assessed

Fisher’s exact test applied. Statistical significance is marked with *

Tumor size changes on T2-weighted images were evaluated according to the RECIST guidelines, placing all patients into either the partial response group (*n* = 11) or the stable disease group (*n* = 11). Fisher’s exact test revealed no significant association between survival status and RECIST groups (*p* = 0.183) (Table 2).

**Table 2:** RECIST^a^ score and status

	Survivors (*n* (%))	Non-survivors (*n* (%))
Partial response	6 (55)	5 (45)
Stable disease	2 (18)	9 (82)

^a^Response evaluation criteria in solid tumors

Fisher’s exact test applied, no correlation (*p* = 0.183)

**Fig. 1:**
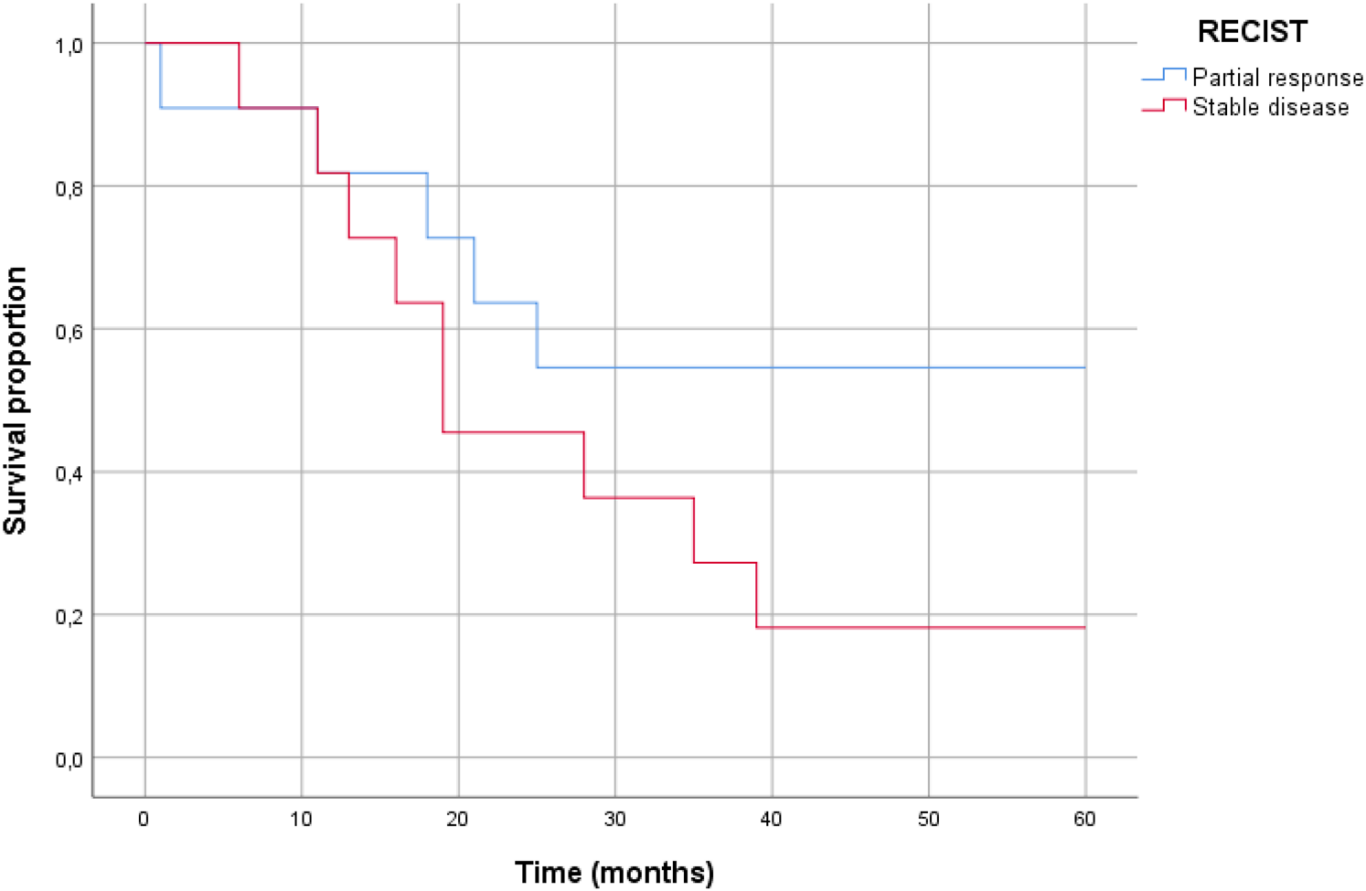
Kaplan Meier survival plot of all patients separated by RECIST (response evaluation criteria in solid tumors) group. No significant difference in survival between the two groups (log rank *p* = 0.15)

The median survival time for the partial response group could not be determined, as over half were still alive at the end of the follow-up period. For the stable disease group, the median survival time was 19 months (interquartile range 13–39 months). There was no significant difference in survival time between the two groups (*p* = 0.150) (Fig. 1).

A comparison of the median SUV_max_, ADC_b0_, and ADC_b50_ values, as well as the changes between the two scans for survivors and non-survivors, showed a significantly lower post-NT SUV_max_ among the long-term survivors (*p* = 0.024) (Table 3). No other statistically significant differences were found between the two groups regarding other median values and differences.

**Table 3:** A comparison of medians of SUV_max_ and ADC-values, survivors versus non-survivors

	Survivors	Non-survivors	*p* value
^18^F-FDG PET			
Pre-NT SUV^a^_max_	9.87 (7.37–16.87)	12.51 (10.48–14.89)	0.664
Post-NT SUV^a^_max_	5.81 (4.41–6.27)	8.37 (7.04–13.25)	0.016*
∆SUV_max_ (%)	−49.75 (−66.98 to −22.78)	−23.65 (−45.13 to 2.48)	0.082
∆ADC_b0_			
Pre-NT ADC^b^	2.04 (1.62–2.13)	1.8 (1.60–2.18)	0.714
Post-NT ADC^b^	1.75 (1.53–2.04)	1.96 (1.78–2.13)	0.360
∆ADC (%)	1.30 (−5.70 to 10.50)	1.70 (−3.00 to 16.08)	0.689
∆ADC_b50_			
Pre-NT ADC^b^	1.77 (1.24–2.04)	1.68 (1.44–1.90)	1
Post-NT ADC^b^	1.86 (1.63–2.03)	1.85 (1.34–1.98)	0.360
∆ADC (%)	16.00 (2.20–33.70)	−1.90 (−7.83 to 16.00)	0.128

Mann–Whitney *U* test applied

Interquartile range is indicated in the parenthesis

^a^Standard Uptake Values (SUV) are depicted as g/cc

^b^Apparent Diffusion Coefficient (ADC) values are depicted as value × 10^–3^ mm^2^/s

^*^ Statistically significant values

**Fig. 2:**
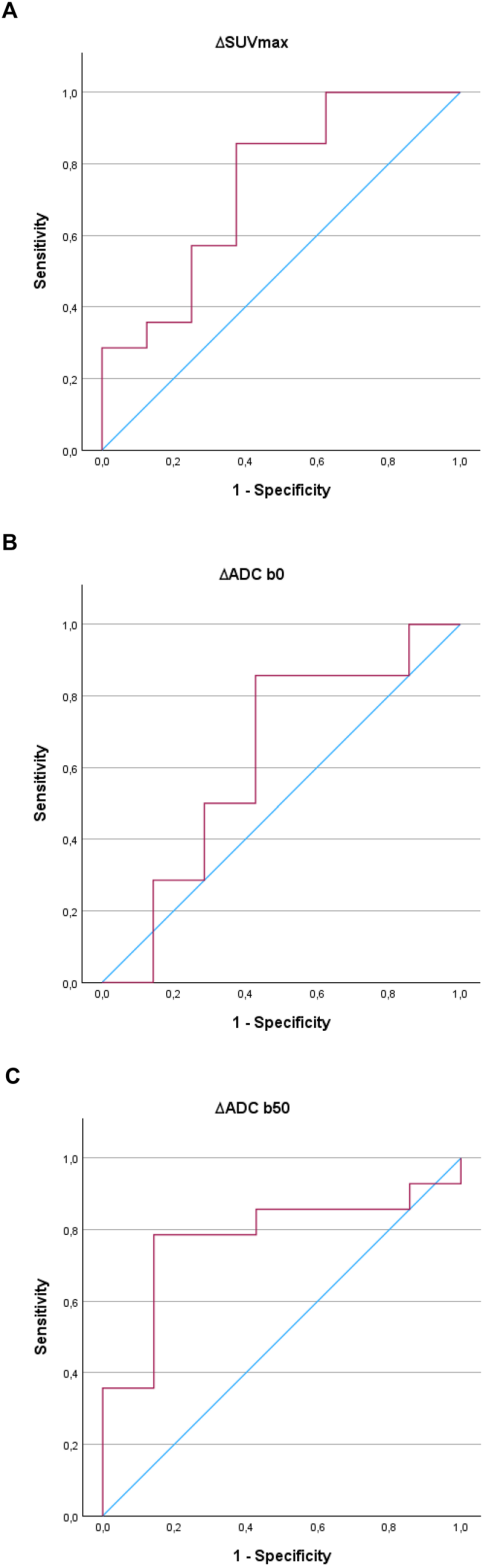

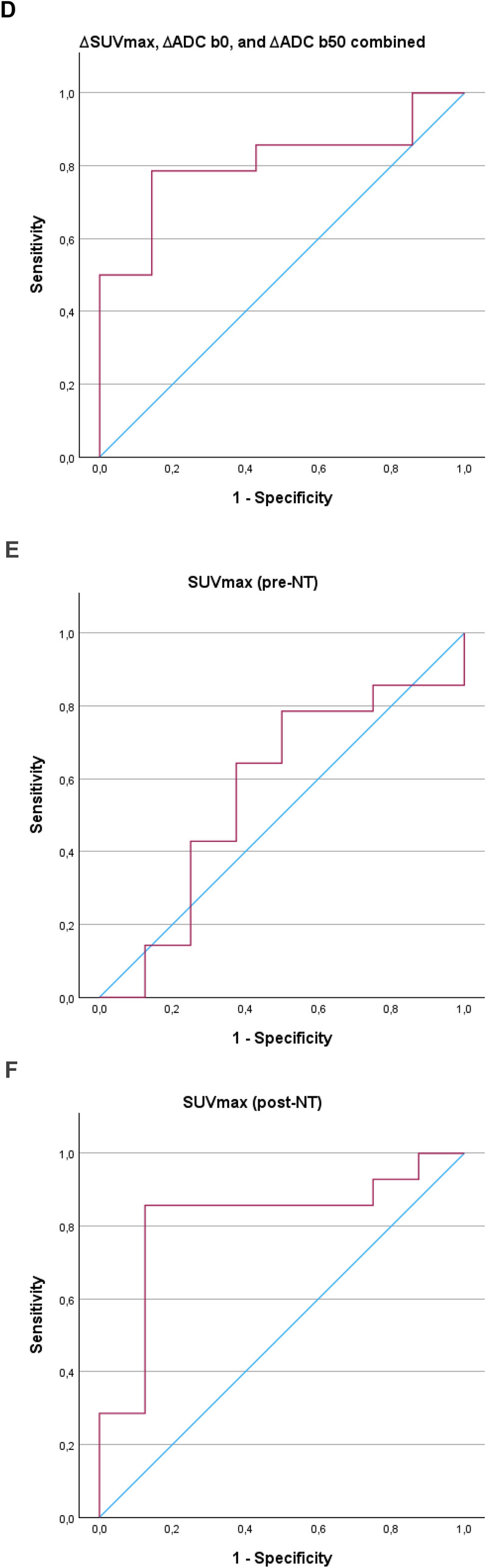

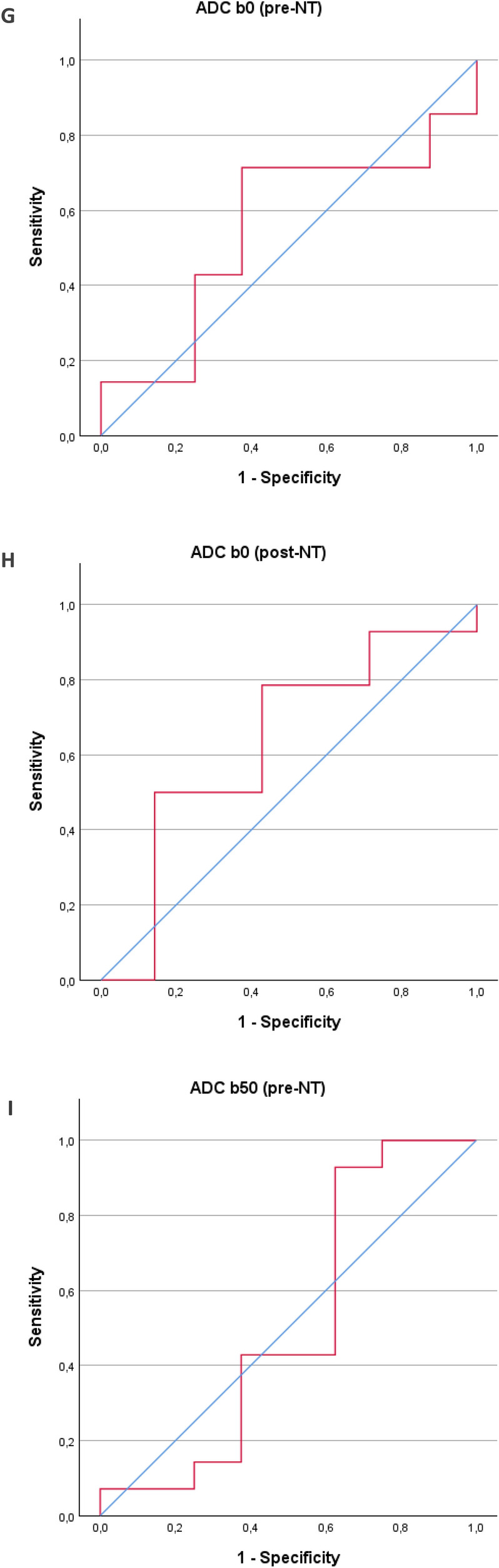

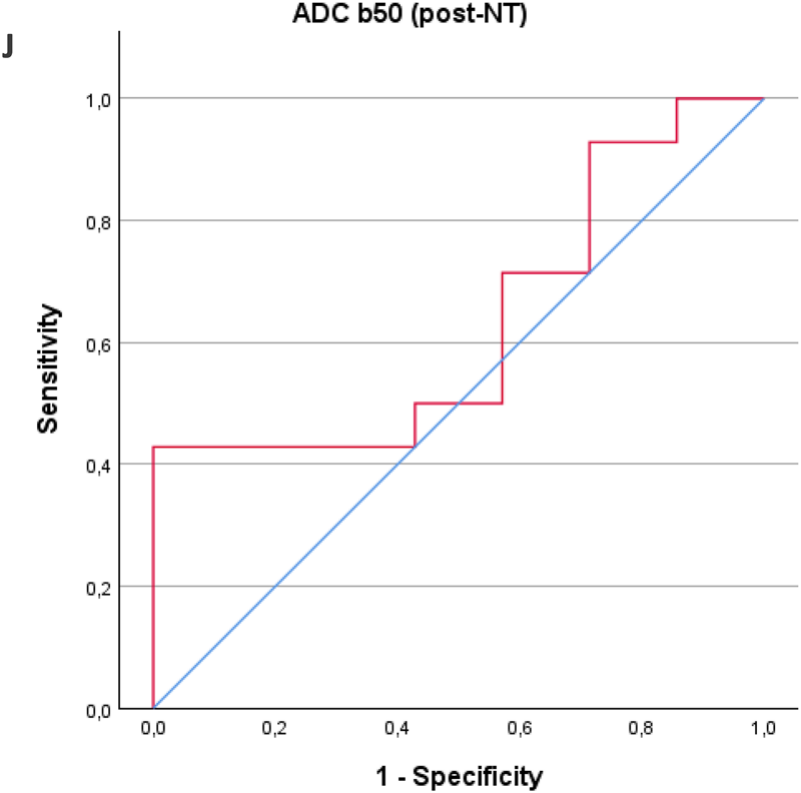
Receiver operating characteristics curves generated from binary logistic regression. **A** Status versus ∆SUV_max_. AUC 0.741 (95% CI 0.515–0.967, *p* = 0.037. **B** Status versus ∆ADC b0. AUC 0.622 (95% CI 0.337–0.907, *p* = 0.400). **C** status versus ∆ADC b50. AUC 0.776 (95% CI 0.563–0.988, *p* = 0.011). **D** Status versus combined parameters. AUC 0.806 (95% CI 0.615–0.997, *p* = 0.002). **E** Status versus pre-NT SUV_max_. AUC 0.56 (95% CI 0.296–0.829, *p* = 0.646). **F** status versus post-NT SUV_max_. AUC 0.81 (95% CI 0.611–1.014, *p* = 0.002). **G** Status versus pre-NT ADC_b0_. AUC 0.55 (95% CI 0.297–0.810, *p* = 0.682). **H** Status versus post-NT ADC_b0_. AUC 0.63 (95% CI 0.361–0.905, *p* = 0.339). **I** status versus pre-NT ADC_b50_. AUC 0.51 (95% CI 0.219–0.799, *p* = 0.952). **J** Status versus post-NT ADC_b50_. AUC 0.63 (95% CI 0.384–0.882, *p* = 0.297). AUC, area under the curve

Receiver operating characteristic (ROC) curves were used to calculate the sensitivity, specificity, and area under the curve (AUC) for ∆SUV_max_, ∆ADC_b0_, and ∆ADC_b50_, and for the single parameters before and after NT, to assess if preoperative scanning could predict long-term survival (Fig. 2). For ∆SUV_max_, the AUC was 0.74, with a sensitivity of 87.5% and specificity of 62.5% (*p* = 0.037). For ∆ADC_b0_, the AUC was 0.62, with a sensitivity of 85.7% and specificity of 57.1% (*p* = 0.400). For ∆ADC_b50_, the AUC was 0.78, with a sensitivity of 78.6% and specificity of 85.7% (*p* = 0.011). The highest AUC of 0.81 was achieved when combining all three parameters, resulting in a sensitivity of 78.6% and specificity of 85.7% (*p* = 0.002). Statistical significance was found for the ∆SUV_max_ curve, ∆ADC b50 curve and the combined curve of ∆SUV_max_, ∆ADC_b0_, and ∆ADC_b50_. For individual measurements, the results were: SUV_max_(pre-NT): AUC = 0.56, sensitivity 78.6%, specificity 50% (*p* = 0.646). SUV_max_(post-NT): AUC = 0.81, sensitivity 85.7%, specificity 87.5% (*p* = 0.002). ADC_b0_(pre-NT): AUC = 0.55, sensitivity 71.4%, specificity 62.5% (*p* = 0.682). ADC_b0_(post-NT): AUC = 0.63, sensitivity 78.6%, specificity 57.1% (*p* = 0.339). ADC_b50_(pre-NT): AUC = 0.51, sensitivity 92.9%, specificity 37.5% (*p* = 0.952). ADC_b50_(post-NT): AUC = 0.63, sensitivity 42.9%, specificity 100% (*p* = 0.279). In the single measurements, only SUV_max_(post-NT) was statistically significant. The AUC, sensibility, and specificity for all measurements are listed in Table 4.

**Table 4:** AUC, sensitivity, specificity, and *p*-values for all ROC curves

	AUC	Sensitivity	Specificity	*p* value
Difference between baseline and evaluation scan				
∆SUV_max_	0.74	87.5%	62.5%	0.037*
∆ADC_b0_	0.62	85.7%	57.1%	0.400
∆ADC_b50_	0.78	78.6%	85.7%	0.011*
∆ All combined	0.81	78.6%	85.7%	0.002*
Pre-NT, single parameters				
SUV_max_	0.56	78.6%	50%	0.646
ADC_b0_	0.55	71.4%	62.5%	0.682
ADC_b50_	0.51	92.9%	37.5%	0.952
Post-NT, single parameters				
SUV_max_	0.81	85.7%	87.5%	0.002*
ADC_b0_	0.63	78.6%	57.1%	0.339
ADC_b50_	0.63	42.9%	100%	0.279

Statistical significance is marked with *

**Fig. 3:**
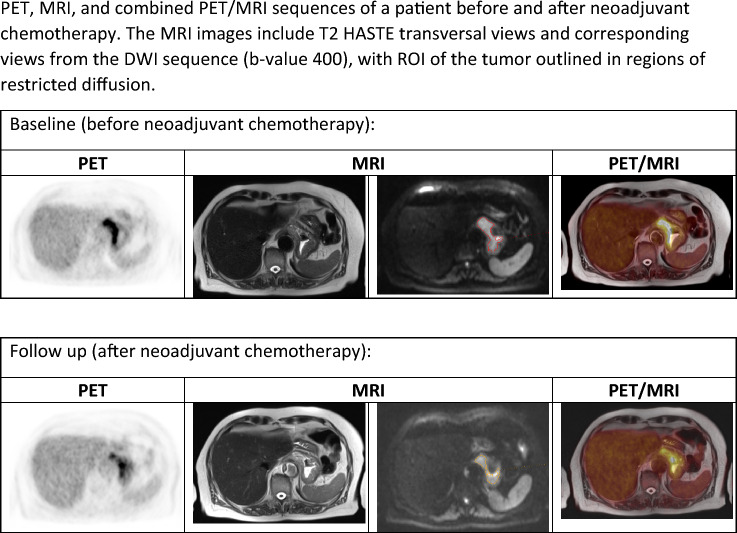
PET, MRI, and combined PET/MRI sequences of a patient before and after neoadjuvant chemotherapy. The MRI images include T2 HASTE transversal views and corresponding views from the DWiI sequence (*b*-value 400), with ROI of the tumor outlined in regions of restricted diffusion

Figure 3 demonstrates MRI, PET, and PET/MRI scans from one patient with hand drawn ROIs. In the supplementary data section, ROC curves can be found for total lesion glycolysis (TLG) and metabolic tumor volume (MTV), as well as changes in TLG and MTV values between the two scans. Comparing the changes for survivors with non-survivors shows no difference between the groups (*p* = 0.095 for TLG, *p* = 0.059 for MTV).

## Discussion

In this five-year follow-up study, we found promising results in identifying long-term survivors among patients with AEG using preoperative PET/MRI evaluation during NT. Furthermore, our study indicates that a lower SUV_max_ after NT may be related to long-term survival.

The RECIST guideline was originally introduced to assess the response to neoadjuvant chemotherapy [14]. However, we found no correlation between survival and RECIST criteria, as the Kaplan–Meier plot showed no significant difference between the partial response and stable disease groups. The lack of significance is likely due to the small sample size of the study. Previous studies have demonstrated that PET/MRI can predict resectability in patients with AEG and offers superior accuracy in assessing treatment response and predict survival in patients with esophageal squamous cell carcinoma [11, 15–17]. However, the prognostic value of simultaneous PET/MRI is not well described for patients with AEG.

The prognostic imaging biomarkers examined in this study include the standard uptake value (SUV) from PET and the apparent diffusion coefficient (ADC) from MRI. Regarding.

SUV, our study found that post-NT SUV_max_ was significantly associated with long-term survival. The utility of SUV_max_ as a parameter is debated; some studies find it useful [18–24], while others do not [25–29]. A 2012 systematic review and meta-analysis found that changes in SUV_max_ on PET scans performed prior to NT and two weeks after initiation could identify patients with AEG having a more favorable prognosis post-surgery [30].

Regarding ADC, our study identified ∆ADC_b50_ as a predictor of long-term survival, with an AUC of 0.78. While other studies have not linked changes in ADC to survival, they have found that ADC reflects the response to NT in AEG tumors [31]. A 2019 meta-analysis also suggested that ADC could serve as an imaging biomarker to predict NT response in patients with AEG or esophageal squamous cell carcinoma [32].

In this study, combining ∆SUV and ∆ADC parameters, along with SUV_max_ post NT, yielded the highest AUCs of 0.81, making these the most significant predictors of long-term survival. Although these findings underscore the benefit of combining PET and MRI imaging to predict long-term survival, PET/MRI does not demonstrate superiority over PET alone. Other studies have investigated the potential benefit of combining these parameters in various cancers, with mixed results [33–35]. However, this is the first study to include AEG patients.

PET/MRI has demonstrated comparable or superior performance to PET/CT in cancer diagnostics, response assessment, and surveillance [17, 36]. Despite the promise of PET/MRI, its longer acquisition times and higher costs have hindered its adoption as a standard of care [37, 38]. Studies have found the per-examination cost of PET/MRI to be about 50% higher [39] and the duration of the typical PET/MRI protocol to be roughly twice as long, with a weekly throughput about five times lower than that of PET/CT [40].

Our results indicate that combining different imaging modalities, such as PET/MRI, cannot only evaluate treatment response but may also predict long-term survival.

Considerable discussion and anticipation exist regarding the potential for total lesion glycolysis (TLG) to replace SUV_max_ and SUV_peak_, which have traditionally been the most widely used metrics [41]. The challenge with TLG is the necessity to select a threshold, and various thresholds are employed, either fixed or as a percentage of SUV_max_ (commonly 40–50%) [42]. This introduces both intra- and inter-observer variation when using metabolic tumor volume (MTV) and TLG. The advantage of SUV_max_ is its extreme reproducibility, as well as its speed and ease of measurement, making it widely used in studies and easily implementable in routine practice. Our study comes with a few limitations. The sample size remains a key limiting factor, increasing the risk of type I and II errors. In addition, evaluation scans were done three weeks after NT initiation, meaning that more accurate response evaluations of who have responded well or poorly at a later point might have missed detection. On the other hand, this study also presents a few strengths. For one, the Danish patient registry's granularity allows for a comprehensive patient history insight, making follow-up accurate and easily doable. Also, this study had more than five years of follow-up, with no patients lost to follow-up.

In conclusion, our results indicate that PET/MRI and PET alone have an equally high accuracy of predicting long-term survivors among AEG patients. Though our results are promising, larger studies are needed to validate and further explore the use in a clinical context.
